# Edge-Terminated AlGaN/GaN/AlGaN Multi-Quantum Well Impact Avalanche Transit Time Sources for Terahertz Wave Generation

**DOI:** 10.3390/nano14100873

**Published:** 2024-05-17

**Authors:** Monisha Ghosh, Shilpi Bhattacharya Deb, Aritra Acharyya, Arindam Biswas, Hiroshi Inokawa, Hiroaki Satoh, Amit Banerjee, Alexey Y. Seteikin, Ilia G. Samusev

**Affiliations:** 1 Department of Electronics and Communication Engineering, Supreme Knowledge Foundation Group of Institutions, Mankundu, Chandannagar 712139, India; monisha.ghosh@skf.edu.in; 2 Department of Mining Engineering, Kazi Nazrul University, Asansol 713340, India; arindam.biswas@knu.ac.in; 3 Department of Electrical Engineering, RCC Institute of Information Technology (RCCIIT), Canal Road, Beliaghata, Kolkata 700015, India; shilpi.bhattacharya@rcciit.org.in; 4 Department of Electronics and Communication Engineering, Cooch Behar Government Engineering College, Harinchawra, Ghughumari, Cooch Behar 736170, India; aritra.acharyya@cgec.org.in; 5 Centre for IoT and AI Integration with Education-Industry-Agriculture, Kazi Nazrul University, Asansol 713340, India; 6 Research Institute of Electronics, Shizuoka University, Hamamatsu 4328011, Japan; inokawa.hiroshi@shizuoka.ac.jp; 7 Microsystem Design-Integration Lab, Physics Department, Bidhan Chandra College, Asansol 713303, India; 8 Computation Biophysics Group, Amur State University, Blagoveshchensk 675027, Russia; seteikin@mail.ru; 9 Research and Education Center for Fundamental and Applied Photonics & Nanophotonics, Immanuel Kant Baltic Federal University, Kaliningrad 236000, Russia

**Keywords:** AlGaN, edge-termination, GaN, IMPATT, multi-quantum well, Schottky barrier, SDR, terahertz

## Abstract

In our pursuit of high-power terahertz (THz) wave generation, we propose innovative edge-terminated single-drift region (SDR) multi-quantum well (MQW) impact avalanche transit time (IMPATT) structures based on the Al*_x_*Ga_1−*x*_N/GaN/Al*_x_*Ga_1−*x*_N material system, with a fixed aluminum mole fraction of *x* = 0.3. Two distinct MQW diode configurations, namely *p^+^-n* junction-based and Schottky barrier diode structures, were investigated for their THz potential. To enhance reverse breakdown characteristics, we propose employing mesa etching and nitrogen ion implantation for edge termination, mitigating issues related to premature and soft breakdown. The THz performance is comprehensively evaluated through steady-state and high-frequency characterizations using a self-consistent quantum drift-diffusion (SCQDD) model. Our proposed Al_0.3_Ga_0.7_N/GaN/Al_0.3_Ga_0.7_N MQW diodes, as well as GaN-based single-drift region (SDR) and 3C-SiC/Si/3C-SiC MQW-based double-drift region (DDR) IMPATT diodes, are simulated. The Schottky barrier in the proposed diodes significantly reduces device series resistance, enhancing peak continuous wave power output to approximately 300 mW and DC to THz conversion efficiency to nearly 13% at 1.0 THz. Noise performance analysis reveals that MQW structures within the avalanche zone mitigate noise and improve overall performance. Benchmarking against state-of-the-art THz sources establishes the superiority of our proposed THz sources, highlighting their potential for advancing THz technology and its applications.

## 1. Introduction

The terahertz (THz) frequency range, often referred to as the “terahertz-gap”, has become a focal point of research and innovation due to its immense potential for a wide range of applications. This spectral region, situated between the millimeter wave and infrared bands, has garnered significant attention in recent years for its promising capabilities. The exploration of the THz-gap has opened up new avenues in fields such as imaging, astronomy, spectroscopy, industrial quality inspection, medical diagnostics, pharmaceutical analysis, and bio-sensing. Researchers have been diligently working to harness the unique properties of THz radiation for diverse practical applications. Among the various THz sources that have demonstrated substantial potential, several state-of-the-art devices have emerged. These include the carcinotron, folded waveguide sources, backward wave oscillators (BWOs), quantum cascade lasers (QCLs), high electron mobility transistors (HEMTs), planar Schottky barrier diode multipliers, and harmonic oscillator arrays [1,2,3,4,5,6,7,8,9,10,11]. These sources have found utility across a wide spectrum of applications, showcasing the versatility and promise of THz technology [12,13,14,15,16,17,18,19]. 

While a plethora of THz sources exist, solid-state devices based on wide bandgap materials, such as Gallium Nitride (GaN) and Silicon Carbide (SiC), have risen to prominence as the most potent contenders in terms of THz power output and DC to THz conversion efficiency [20,21,22]. Gallium Nitride, in particular, stands out due to its superior electronic and thermal properties, making it the material of choice for fabricating high-power, high-efficiency THz sources. However, despite the potential of GaN-based impact avalanche and transit time (IMPATT) diodes for THz wave generation, a significant impediment has hampered their widespread adoption. This obstacle arises from the high contact resistivity encountered in metal–*p*^+^-GaN ohmic contacts. To date, the lowest experimentally achieved contact resistivity for such contacts stands at 10^−4^–10^−3^ Ω cm^2^ [23,24], which poses a substantial challenge. This elevated contact resistivity results in an increase in parasitic series resistance within the diode, diminishing its negative resistance at THz frequencies [25]. The consequent high series resistance constitutes the primary limitation to harnessing THz power from GaN IMPATT sources. In a concerted effort to circumvent the detrimental effects of metal–*p*^+^-GaN contacts in IMPATT diode structures for THz wave generation, researchers have explored alternative designs. One such alternative is the Schottky barrier IMPATT structure [26,27]. In 2023, Khan et al. [28] introduced a lateral Schottky barrier IMPATT structure known as the HEM-ATT diode. This innovative device combines elements of a HEMT structure based on AlGaN/GaN two-dimensional electron gas (2-DEG) with a Schottky barrier single-drift region (SDR) avalanche transit-time (ATT) structure [28]. The resulting HEM-ATT diode exhibited remarkable capabilities, delivering 300 mW of continuous peak power with an 11% conversion efficiency at 1.0 THz. This represents a significant leap forward compared to the achievable THz power output and efficiency of conventional double-drift region (DDR) IMPATT sources [28].

Building upon this promising progress, our research endeavors to further explore the potential for high-power, high-efficiency THz wave generation using GaN IMPATT sources. We focus on the investigation of multi-quantum well (MQW) IMPATT sources based on the AlGaN/GaN material system. In particular, we examine the following three distinct IMPATT structures: Al_0.3_Ga_0.7_N/GaN/Al_0.3_Ga_0.7_N MQW SDR (IMPATT-1), flat GaN SDR (IMPATT-2), and Al_0.3_Ga_0.7_N/GaN/Al_0.3_Ga_0.7_N MQW Schottky barrier IMPATT diodes. These diodes were meticulously designed and optimized for biasing to operate around a 1.0 THz frequency. The challenge of premature and soft breakdown in the IMPATT diodes was effectively mitigated through the implementation of a well-suited edge termination method, which integrated mesa etching and nitrogen ion implantation techniques [29,30]. Our investigation not only delves into the steady-state and dynamic performance of these structures but also explores their avalanche noise characteristics at THz frequencies. To provide a comprehensive perspective, we compare the THz performance of these novel structures with a previously proposed MQW DDR IMPATT source (IMPATT-4) based on the 3C-SiC/Si material system [31].

Moreover, we benchmark the performance of our proposed structures against that of state-of-the-art THz sources [1,2,3,4,5,6,7,8,9,10,11], providing insights into their relative capabilities. In conclusion, we observe that the Al_0.3_Ga_0.7_N/GaN/Al_0.3_Ga_0.7_N MQW Schottky barrier IMPATT source surpasses all other potential THz sources in terms of THz power delivery and DC to THz conversion efficiency. The incorporation of Al_0.3_Ga_0.7_N/GaN/Al_0.3_Ga_0.7_N MQWs enhances the avalanche noise performance of the source at THz frequencies, making it a standout candidate for future THz applications. Our research, thus, contributes to the ongoing exploration of the THz-gap and the development of cutting-edge THz technology with substantial implications for various fields of science and industry.

## 2. Structures, Design and Fabrication Possibilities

**Figure 1:**
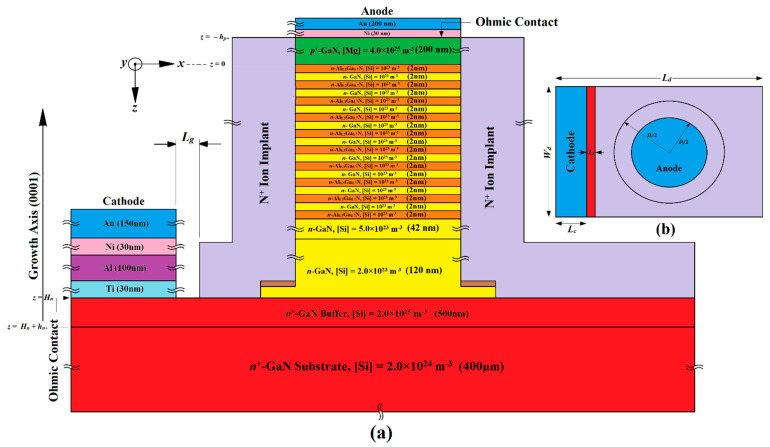
(**a**) Cross-sectional structure diagram of the Al_0.3_Ga_0.7_N/GaN/Al_0.3_Ga_0.7_N MQW SDR IMPATT diode (IMPATT-1); (**b**) top view of the device structure.

**Figure 2:**
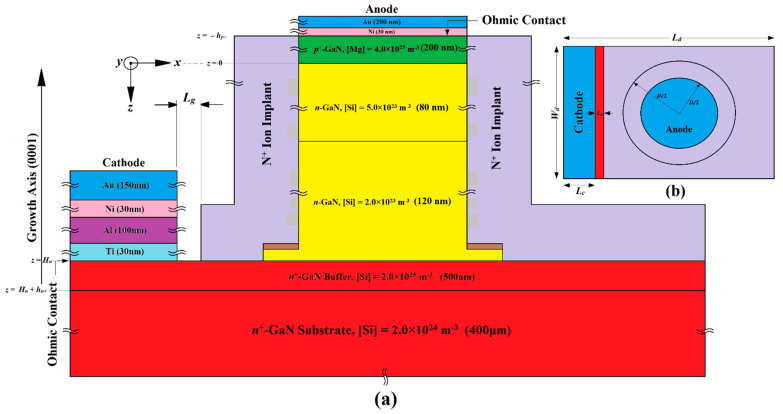
(**a**) Cross-sectional structure diagram of the GaN SDR IMPATT diode (IMPATT-2); (**b**) top view of the device structure.

**Figure 3:**
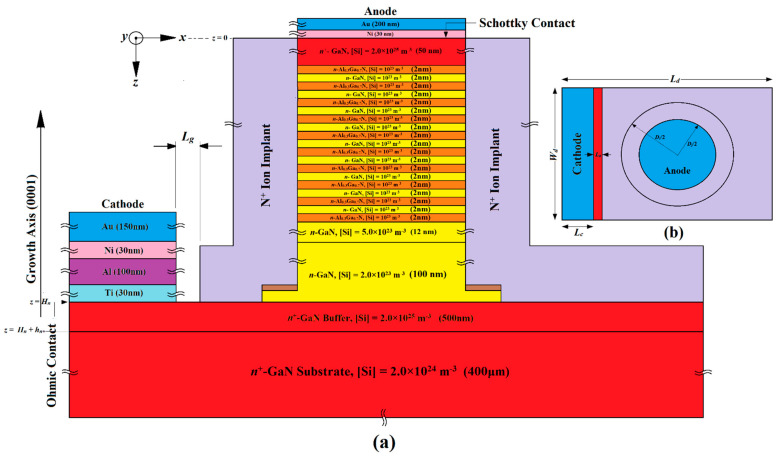
(**a**) Cross-sectional structure diagram of the Al_0.3_Ga_0.7_N/GaN/Al_0.3_Ga_0.7_N MQW Schottky barrier IMPATT diode (IMPATT-3); (**b**) top view of the device structure.

This section provides a detailed exploration of the proposed device structures, design principles, and possible fabrication processes of three distinct IMPATT diodes (referred to as IMPATT-1, IMPATT-2, and IMPATT-3). It also includes a comparison with a previously documented 3C-SiC/Si/3C-SiC MQW DDR diode (IM-PATT-4) engineered for operation at 1.0 THz [31]. Initially, the thickness of the *n*-type active region (*H_n_*) for each diode type was determined using an empirical relation based on the saturation drift velocity of electrons (*v_sn_*) in the base semiconductor (GaN) and the design frequency (*f_d_*) (*H_n_* = 0.37*v_sn_*/*f_d_* [32]). The diodes were meticulously designed to maintain quasi-Read doping profiles, ensuring a narrow width for the avalanche layer, which greatly enhances their high-frequency performance and reduces noise levels [33,34]. The doping profiles for IMPATT-1, IMPATT-2, and IMPATT-3 follow specific concentration patterns, such as low–high–low (lo–hi–lo), high–low (hi–lo), and high–low–high–low (hi–lo–hi–lo) respectively. In IMPATT-1 and IMPATT-3, a total of nine cycles of Al_0.3_Ga_0.7_N/GaN/Al_0.3_Ga_0.7_N quantum wells were incorporated to confine charge multiplication phenomena within a short region due to the quantum confinement of electrons in the bound states of the quantum wells, enhancing DC to THz conversion efficiency and minimizing avalanche noise [35]. The thickness of both the barrier (Al_0.3_Ga_0.7_N) and well (GaN) layers was chosen to be 2 nm in order to form the 38 nm long MQW structure in both IMPATT-1 and IMPATT-3. The doping levels of both barriers and well layers were kept low (10^23^ m^−3^) in those diode structures. Two drift layers, drift layer-2 (DL-2) and drift layer-1 (DL-1), facilitated electron drift motion under reverse bias. The IMPATT-2 compensated for the absence of MQW layers by extending DL-2, maintaining a consistent active layer thickness (*H_n_*) across all diodes. For IMPATT-3, an additional highly doped (~10^25^ m^−3^) *n*^+^-GaN layer was added over the MQW layers to ensure a quality Schottky anode contact. Following the initial determination of layer thicknesses and doping levels, large-signal simulations based on the self-consistent quantum drift-diffusion (SCQDD) model were utilized to optimize these parameters within a specific range of bias current density (for which the diodes exhibited negative resistance), which were subject to achieve maximum DC to THz conversion efficiency [20,21,22,31]. Ultimately, Figure 1, Figure 2 and Figure 3 provide the optimal values for the thickness and doping concentrations of all layers in IMPATT-1, IMPATT-2, and IMPATT-3, respectively.

The cross-sectional structure diagram and top view of the Al_0.3_Ga_0.7_N/GaN/Al_0.3_Ga_0.7_N MQW IMPATT diode (IMPATT-1) are shown in Figure 1a,b. The proposed fabrication process began with a 400 µm thick, 4-inch diameter *n*^+^-GaN substrate with a doping concentration of 2.0 × 10^24^ m^−3^, on which around 500–600 diodes or more could be simultaneously fabricated. The Al_0.3_Ga_0.7_N/GaN/Al_0.3_Ga_0.7_N quantum wells (MQWs) could be grown using molecular beam epitaxy (MBE) on the *n*^+^-GaN substrate along the (0001) direction. To reduce growth stress and for cathode contact formation, a 500 nm thick *n*^+^-GaN buffer layer had to be grown at a substrate temperature of 720 °C and doped with Si at a concentration of 2.0 × 10^25^ m^−3^. Next, two *n*-GaN drift layers (DL-1 and DL-2) were grown on the buffer layer, with DL-1 being 120 nm thick and doped with Si at a concentration of 2.0 × 10^23^ m^−3^ and DL-2 being 42 nm thick with a higher doping concentration of 5.0 × 10^23^ m^−3^. Approximately 9–10 cycles of Al_0.3_Ga_0.7_N/GaN/Al_0.3_Ga_0.7_N quantum wells were grown on DL-2, with 2 nm thick GaN wells and Al_0.3_Ga_0.7_N barrier layers. These barriers and well layers must be doped with Si at a concentration of 1.0 × 10^23^ m^−3^. The diode features a low_(MQWs)_–high_(GaN)_–low_(GaN)_ doping density profile, which confines charge multiplication within a narrow region and reduces the effective avalanche zone under reverse bias, improving DC to THz efficiency and avalanche noise performance [31,35]. After the growth of MQWs, a 300 nm thick *p*^+^-GaN layer had to be grown on the final Al_0.3_Ga_0.7_N layer to form the *p^+^-n* junction. This layer had to be doped with Mg at a concentration of 4.0 × 10^25^ m^−3^. The hard-masking SiO_2_, electron beam lithography and Cl_2_-based inductively coupled plasma reactive ion etching (ICP-RIE) had to be used to etch around 500 nm of GaN and AlGaN layers, forming a diode mesa structure with a 10 µm circular cross-section. Anode contact can be formed by the vacuum evaporation of Ni (30 nm) and Au (200 nm) over the *p*^+^-GaN layer. Cathode ohmic contact can be formed using a thermal evaporation process involving Ti (30 nm), Al (100 nm), Ni (30 nm), and Au (150 nm) layers over the *n*^+^-GaN buffer layer. Next, the nitrogen ion implantation must be performed to create a high-resistivity edge termination layer. A ring-shaped photo-resist mask (outer diameter *D_i_* = 10 µm and inner diameter *D_j_* = 5 µm) can be used for implantation. Multiple implantation steps must be conducted with increasing energy and doses to achieve a flat defect density profile. A 50 nm section of the *n*-GaN layer (DL-1) must be kept partially compensated (the brown region in Figure 1a) to optimize the electric field distribution at the edges [29,30]. 

The decision to use a multi-layered composite metal contact for the cathode required thorough investigation. Aluminum (Al) emerged as a prime candidate due to its suitability for forming metal–*n*^+^-GaN ohmic contact. To achieve a low-resistivity single-layer Al~*n*^+^-GaN ohmic contact, brief annealing at moderate temperatures (around 550–575 °C) is necessary. Prolonged annealing at higher temperatures (>900 °C) leads to Al oxidation and the formation of a thin Al_2_O_3_ layer at the Al~*n*^+^-GaN interface, significantly increasing contact resistivity. Another viable option for achieving low-resistivity metal–*n*^+^-GaN ohmic contact is titanium (Ti). However, producing a highly conductive single-layer Ti~*n*^+^-GaN ohmic contact requires high-temperature annealing (around 900–950 °C). At these temperatures, a thin TiN layer forms at the Ti~*n*^+^-GaN interface, enhancing doping by increasing N-vacancies and consequently increasing the tunneling current and reducing contact resistivity [36,37]. However, Ti is prone to oxidation even at room temperature. Therefore, instead of using a single-layer Al or Ti contact, a composite Ti (contact layer)/Al (cap layer) ohmic contact can be deposited on the *n*^+^-GaN layer and annealed at 900 °C for 30 s to achieve very low-resistivity, stable, and high-performance ohmic contact [38]. Additionally, depositing a less resistive and less prone-to-oxidation gold (Au) layer on the Ti/Al layer significantly improves its performance [39]. To prevent high-energy Au atoms from penetrating through the Al layer at high temperatures, a nickel (Ni) barrier layer must be deposited between the Al and Au layers [38]. Ultimately, Ti (contact layer)/Al (over-layer)/Ni (barrier layer)/Au (cap layer)-based multilayer ohmic contact proves to be the most favorable for achieving low-contact resistivity and stable cathode contact, especially for THz IMPATT structures.

The fabrication procedures for GaN SDR IMPATT (IMPATT-2) and Al_0.3_Ga_0.7_N/GaN/Al_0.3_Ga_0.7_N MQW Schottky barrier (IMPATT-3) diodes shown in Figure 2 and Figure 3, respectively, are similar to that of Al_0.3_Ga_0.7_N/GaN/Al_0.3_Ga_0.7_N MQW (IMPATT-1) diode (Figure 1). The key difference in GaN SDR IMPATT (IMPATT-2) diode fabrication is the substitution of the Al_0.3_Ga_0.7_N/GaN/Al_0.3_Ga_0.7_N MQWs layers with the *n*-GaN DL-2 extended up to an 80 nm thickness and resulting in the overall high–low doping profile of the active region of the diode (formed by DL-2 and DL-1). In the Al_0.3_Ga_0.7_N/GaN/Al_0.3_Ga_0.7_N MQW Schottky barrier (IMPATT-3) diode, a 50 nm thick *n*^+^-GaN layer doped with Si dopants of dosage 2.0 × 10^25^ m^−3^ had to be grown over the final Al_0.3_Ga_0.7_N barrier layer with higher doping concentrations than the MQW layers. There was no *p^+^-n* junction in IMAPTT-3, and instead, a Schottky contact layer was formed using Ni (30 nm) and Au (200 nm) deposition. 

A previously reported diode structure (referred to as IMPATT-4) designed for operation at 1.0 THz was considered for comparative analysis with IMPATT-1, IMPATT-2, and IMPATT-3 in terms of their THz performance [31,35]. The IMPATT-4 adopts a *p^+^-p-n-n^+^* configuration, and one can find detailed information about its structure in references [31,34]. In IMPATT-4, there are 9–10 cycles of 3C-SiC/Si/3C-SiC multiple quantum wells (MQWs) symmetrically positioned on both sides of the *p-n* junction [31]. The 3C-SiC (barrier) and Si (well) layers in these MQWs have thicknesses of 2 nm each, with doping concentrations maintained at 20.0 × 10^23^ m^−3^ to achieve a low–high doping density profile. The IMPATT-4 shares the same cross-sectional diameter as IMPATT-1, IMPATT-2, and IMPATT-3, which is *D_j_* = 5 µm.

Lattice-mismatched systems, such as Al_0.3_Ga_0.7_N/GaN/Al_0.3_Ga_0.7_N-based MQW structures, generate dislocations once the strain energy surpasses a critical point, releasing the accumulated strain energy as the film thickness increases. These dislocations significantly degrade the high-frequency performance of MQW IMPATT devices, which is practically inevitable in highly lattice-mismatched epitaxy. The density of dislocations and other defects plays a crucial role in determining the material system’s performance. Researchers have recently developed techniques to enhance the quality of III–V materials during heteroepitaxial growth, similar to the growth of the MQW structure discussed here. Some of these techniques are noteworthy. Epitaxy and layer lift-off techniques have facilitated the production of a wide range of two-dimensional materials and three-dimensional single-crystalline freestanding thin films with diverse optical functionalities [40]. These films feature van der Waals (vdW) interfaces suitable for photonic vdW integration. Physical assembly utilizing vdW interactions removes the constraints of epitaxial lattice-matching, enabling the combination of dissimilar materials with attractive optoelectronic properties but radically different crystal structures. Various prefabricated vdW building blocks can be assembled into novel hetero-integrated photonic architectures and hybrid vdW heterostructures to prototype new devices and explore exotic nanophotonic phenomena at mixed-dimensional vdW interfaces [40]. Furthermore, in 2020, Hoon Bae et al. introduced a unique mechanism for relaxing misfit strains in heteroepitaxial films, enabling effective lattice engineering [41]. They observed that heteroepitaxy on graphene-coated substrates allows for the spontaneous relaxation of the misfit strain due to the slippery graphene surface while achieving single-crystalline films by reading the atomic potential from the substrate. This spontaneous relaxation technique could revolutionize the monolithic integration of largely lattice-mismatched systems, enhancing and broadening the functionality of semiconductor devices for advanced electronics and photonics.

The fabrication of Al_0.3_Ga_0.7_N/GaN/Al_0.3_Ga_0.7_N MQW structures presents several experimental challenges crucial to addressing optimal device performance. One significant challenge involved managing the strain induced by lattice mismatch between layers within the MQW structure. For instance, the growth of AlGaN on GaN can cause strain accumulation, affecting crystal quality and device performance. Utilizing proper strain engineering techniques becomes essential to mitigate these effects. Another challenge is the presence of high dislocation density in epitaxial layers, particularly at the interfaces, which can degrade material quality and electronic properties. Techniques like strain relaxation methods and epitaxial growth optimization are vital for reducing dislocation density. Some recently developed techniques to deal with these challenges have already been mentioned in the earlier paragraph [40,41]. Ensuring uniformity in material composition and thickness across epitaxial layers is also critical for consistent device performance [42]. Any variations in material properties can lead to non-uniform device characteristics and reduced yield. Additionally, maintaining a smooth surface morphology is crucial to ensure good interface quality and minimize scattering effects, which are vital for high-performance MQW THz diodes [43]. Lastly, accurate characterization techniques such as high-resolution X-ray diffraction, transmission electron microscopy, and atomic force microscopy are necessary to assess the crystal quality, composition, and structural properties of the MQW layers. These techniques play a significant role in addressing the challenges associated with material preparation for Al_0.3_Ga_0.7_N/GaN/Al_0.3_Ga_0.7_N MQW structures.

It is clear that the THz and noise performance of the proposed device structures are highly dependent on the material quality. Therefore, performing thorough material characterization of the device’s structures is essential to evaluate the crystal quality of the fabricated devices. The determination of Al content and crystal quality of the epilayers can be accomplished by employing a high-resolution X-ray diffractometer (such as Brucker D8 DISCOVER, Billerica, MA, USA). For a detailed examination of the device cross-section, particularly the multi-quantum well (MQW) region, it is recommended that a high-resolution scanning transmission electron microscope (HR-STEM) is utilized. The mesa and electrodes of the device structures can be examined using an optical microscope (like Nikon ECLIPSE LV15ONA, Tokyo, Japan). To obtain current–voltage (I–V) curves, the PDA FS-Pro 380 semiconductor (Primarius, Hsinchu, Taiwan) analyzer is suitable. A comprehensive report detailing the results of the full-structure device characterization will be presented in an upcoming presentation.

The characterization of the proposed diode structures in the THz regime presents challenges due to the lack of a standard THz power measuring tool. To assess the THz performance of the proposed MQW sources, two potential THz power measurement techniques can be employed. The first option involves utilizing a traceable THz power measurement technique developed by Steiger et al. in 2013, which is capable of measuring radiated power within the 1–5 THz range [44]. Alternatively, a more precise method employs a pyroelectric thin-film detector based on the polyvinylidene fluoride (PVDF) film for radiated THz power measurement [45]. Additionally, the THz noise characterization of the proposed MQW sources can be achieved by combining device impedance measurement techniques at THz frequencies with experimental measurements of the diodes’ intrinsic response time [46]. Furthermore, low-frequency noise resulting from fluctuations in bias current and device junction temperature can be assessed using the technique described in ref. [46].

## 3. Simulation Technique

The authors employed a self-consistent quantum drift diffusion (SCQDD) model in their research to perform the steady-state DC simulations of IMPATT diodes operating under reverse bias [31]. This SCQDD model was developed by coupling classical drift diffusion (CLDD) equations with Schrödinger’s time-independent equations corresponding to electrons in the conduction band and holes in the valence band [31]. The CLDD equations are presented below:

Poisson’s equation:

(1) $$\frac{d}{dz}\left(-\epsilon \frac{dV}{dz}+P_{t}\right)=q\left(\gamma_{p}p-\gamma_{n}n+N\right),$$

Steady-state continuity equations:

(2) $$\frac{dJ_{r}}{dz}=\pm q\left(\sum_{\Xi }G_{r\left(\Xi \right)}-R_{r}\right),$$

Current density equations:

(3) $$J_{r}=-q\left\{\gamma_{r}r\mu_{r}\pm D_{r}\frac{d\left(\gamma_{r}r\right)}{dz}\right\}.$$

The Equations (1)–(3) included various parameters, where *q* = 1.6 × 10^−19^ C represents the unit electronic charge. The spatially dependent parameters, denoted as *r* ∈ {*p*, *n*}, refer to hole and electron concentrations. The functions *G_r_*_(Ξ)_ signify the generation rates of holes and electrons, accounting for avalanche multiplication (Ξ ≡ AV), band-to-band tunneling (Ξ ≡ BBT), trap-assisted tunneling (Ξ ≡ TAT), and thermionic field emission (Ξ ≡ TFE). The electron generation rate due to TFE in the Schottky barrier diode can be expressed as follows [47,48]:

(4) $$G_{TFE}\left(\xi \right)=\frac{A^{*}T\hbar \xi }{k_{B}}{\left(\frac{\pi }{2m_{n}^{*}k_{B}T}\right)}^{\frac{1}{2}}\int_{z=0}^{z=H_{n}}exp\left[-\frac{1}{k_{B}T}\left(\phi_{B}-\frac{{\left(e\hbar \xi \right)}^{2}}{24m_{n}^{*}{\left(k_{B}T\right)}^{2}}\right)\right],$$

where the barrier height is given by

(5) $$\phi_{B}=k_{B}Tln\left(\frac{A^{*}T^{2}}{J_{ns}}\right).$$

In Equations (4) and (5), $A^{*}=\left(4\pi qm_{n}^{*}k_{B}^{2}/h^{3}\right)$ denotes the effective Richardson constant, ℏ=h/2π represents the normalized Planck’s constant (with Planck’s constant *h* = 6.62 × 10^−31^ J s), *T* symbolizes the temperature in Kelvin (K), *k_B_* = 1.38 × 10^−23^ J K^−1^ is the Boltzmann constant, $m_{n}^{*}$ is the electron effective mass in GaN, ξ=−dV/dz is the electric field and $J_{ns}$ is the reverse saturation current due to electrons. The term related to the electron generation rate due to thermionic field emissions (i.e., $G_{TFE}$) is only present in Equation (2) for the simulation of the Schottky barrier IMPATT (i.e., IMPATT-3) simulation. Additionally, in Equations (1)–(3), *R_r_* represents the Shockley–Hall–Read recombination rates for holes and electrons, *μ_r_* denotes the hole and electron mobility, and *D_r_* corresponds to the diffusion constants associated with holes and electrons. The *γ_r_* parameters, initially set to unity at the first iteration, represent quantum-correction factors for holes and electrons (here, the subscripts *r* ≡ *n* and *r* ≡ *p* correspond to electrons and holes, respectively). Another parameter, *N*(*z*), characterized the space-dependent doping profile of the device. The solutions for the space-dependent electric potential (*V*(*z*)), electron density (*n*(*z*)), hole density (*p*(*z*)), electron current density (*J_n_*(*z*)), and hole current density (*J_p_*(*z*)) under a given biasing condition were obtained by concurrently solving CLDD Equations (1)–(3) using the finite difference method (FDM). The boundary conditions applied at the depletion layer edges, specifically at *p^+^-n* (*z* = 0) and *n-n^+^* (*z* = *H_n_*) interfaces in IMPATT-1 and IMPATT-2, and at (M)-*n^+^* (*z* = 0) and *n-n^+^* (*z = H_n_*) interfaces of IMPATT-3 (where (M)-*n^+^* represents the Schottky contact with M ≡ Au/Ni), are given as follows [30]:

Electric field boundary conditions:

(6) $${\left.\frac{dV}{dz}\right|}_{z=0}={\left.\frac{dV}{dz}\right|}_{z=H_{n}}=0$$

Current density boundary conditions:

(7) $$NJ\left(z=0\right)=\left\{1-\frac{2J_{n}\left(z=0\right)}{J_{p}\left(z=0\right)+J_{n}\left(z=0\right)}\right\},$$

(8) $$NJ\left(z=H_{n}\right)=\left\{\frac{2J_{p}\left(z=H_{n}\right)}{J_{p}\left(z=H_{n}\right)+J_{n}\left(z=H_{n}\right)}-1\right\},$$

where the normalized current density is given by $NJ=\left(J_{p}-J_{n}\right)/\left(J_{p}+J_{n}\right)=\left(J_{p}-J_{n}\right)/J_{0}$ for a given bias current density of $J_{0}=\left(J_{p}+J_{n}\right)$. 

In semiconductor materials belonging to group-III nitrides, such as wurtzite GaN and its binary compound Al*_x_*Ga_1−*x*_N, distinctive polarization properties are observed. These materials inherently possess spontaneous polarization properties even in an unstrained state, leading to strain-independent built-in electrostatic fields [49]. These electric fields, induced by spontaneous polarization, significantly impact the transport and optical characteristics of nanostructures based on group-III nitrides. Conversely, both wurtzite GaN and Al*_x_*Ga_1−*x*_N exhibit piezoelectric polarization fields under strained conditions [49]. In the Al*_x_*Ga_1−*x*_N/GaN/Al*_x_*Ga_1−*x*_N multiple quantum well (MQW) structure, the substantial difference in lattice constants between GaN and Al*_x_*Ga_1−*x*_N results in strain when pseudomorphic growth is present. Consequently, both the active (GaN) and cladding (Al*_x_*Ga_1−*x*_N) layers experience strain, giving rise to piezoelectric polarization fields [49]. The spontaneous polarization aligns with the growth direction, specifically along the (0001) axis. However, piezoelectric polarization can take any direction. In wurtzite nitrides, the growth direction consistently aligns with the polar (0001) axis, inducing piezoelectric polarization along the growth axis due to the presence of non-accommodated in-plane mismatches [49]. The sign of the piezoelectric polarization depends on the type of epitaxial strain, whether tensile or compressive. The electric fields induced by polarization in the well (GaN) and barrier (Al*_x_*Ga_1−*x*_N) layers of the Al*_x_*Ga_1−*x*_N/GaN/Al*_x_*Ga_1−*x*_N MQW structure are expressed as follows:

(9) $${\vec{\xi }}_{w}^{\left(\chi \right)}=\frac{4\pi h_{b}\left({\vec{P}}_{b}^{\left(\chi \right)}-{\vec{P}}_{w}^{\left(\chi \right)}\right)}{\left(h_{b}\epsilon_{w}+h_{w}\epsilon_{b}\right)},$$

(10) $${\vec{\xi }}_{b}^{\left(\chi \right)}=\frac{4\pi h_{w}\left({\vec{P}}_{w}^{\left(\chi \right)}-{\vec{P}}_{b}^{\left(\chi \right)}\right)}{\left(h_{b}\epsilon_{w}+h_{w}\epsilon_{b}\right)},$$

In Equations (9) and (10), *ϵ_w_*_,*b*_ represents the dielectric constants and *h_w_*_,*b*_ denotes the thicknesses of the well and barrier layers. The parameter *χ* ∈ {*sp*, *pz*} distinguishes between spontaneous (*sp*) and piezoelectric (*pz*) polarizations while ${\vec{P}}_{w,b}^{\left(\chi \right)}$ correspond to *χ*-type polarization in the GaN-well and Al*_x_*Ga_1−*x*_N-barrier. Notably, Equations (9) and (10) reveal that when *h_w_ = h_b_*, the relation ${\vec{\xi }}_{w}^{\left(\chi \right)}=-{\vec{\xi }}_{b}^{\left(\chi \right)}$ holds true, which is a condition pertinent to the MQW structure under consideration (*h_w_* = *h_b_* = 2 nm). Consequently, under arbitrary strained conditions, the overall polarization-induced electric field can be expressed as follows:

(11) $${\vec{\xi }}_{p}=\sum_{\chi \in \left\{sp,pz\right\}}{\vec{\xi }}_{b,w}^{\left(\chi \right)}.$$

In the context of the pseudomorphically grown Al*_x_*Ga_1−*x*_N/GaN/Al*_x_*Ga_1−*x*_N MQW structure on a GaN substrate, where *h_w_ = h_b_*, the following relationship is sustained:

(12) $$\sum_{\chi \in \left\{sp,pz\right\}}{\vec{\xi }}_{w}^{\left(\chi \right)}=-\sum_{\chi \in \left\{sp,pz\right\}}{\vec{\xi }}_{b}^{\left(\chi \right)}.$$

Consequently, the determination of the position-dependent total transverse polarization (*P_t_*) in Equation (1) becomes feasible through the relation ${\vec{P}}_{t}=\epsilon {\vec{\xi }}_{p}$, where *ϵ* represents the position-dependent dielectric constant. The spontaneous and piezoelectric polarization components in the Al*_x_*Ga_1−*x*_N cladding layers can be derived from the following equations [49]:

(13) $$P_{b}^{\left(sp\right)}=xP_{AlN}^{\left(sp\right)}+\left(1-x\right)P_{GaN}^{\left(sp\right)},$$

(14) $$P_{b}^{\left(pz\right)}=2S_{xy}\left\{e_{31}-\left(\frac{C_{13}}{C_{33}}\right)e_{33}\right\}.$$

In a given context, where *x* represents the Al mole fraction in Al*_x_*Ga_1−*x*_N, $S_{xy}=\left(a_{GaN}-a_{{Al}_{x}{Ga}_{1-x}N}\right)/a_{{Al}_{x}{Ga}_{1-x}N}$ denotes the strain in the *x*-*y* plane. The piezoelectric constants are represented by *e*_31_ and *e*_33_, while the elastic constants are denoted by *C*_13_ and *C*_33_. The lattice constants of GaN and Al*_x_*Ga_1−*x*_N, $a_{GaN}$ and $a_{{Al}_{x}{Ga}_{1-x}N}$ respectively, are also included. The simulation method incorporates the model developed by Fiorentini et al. [49], where 40% of the polarization charges are compensated by defects and interface charges. Additionally, various material parameters associated with polarization in wurtzite group-III nitrides (GaN and Al*_x_*Ga_1−*x*_N) are sourced from reference [50].

Upon deriving solutions for the CLDD equations in a specific iteration, the 1-D time-independent Schrödinger equations linked to the conduction and valence bands were solved to incorporate the presence of bound states within the quantum wells (QWs). The Schrödinger equations associated with the valence and conduction bands are expressed as follows [30]:

(15) $$-\frac{d}{dz}\hat{z}.\left(\frac{\hbar }{2m_{r}^{*}}\frac{d\psi_{r}^{Z_{r}}}{dz}\hat{z}\right)+E_{V,C}\psi_{r}^{Z_{r}}=E_{r}^{Z_{r}}\psi_{r}^{Z_{r}}.$$

In Equation (15), the effective mass of holes in the valence band and electrons in the conduction band are denoted by $m_{r}^{*}$. The wave-function and eigen-energy state solutions linked to the valence and conduction bands are expressed as $\psi_{r}^{Z_{r}}$ and $E_{r}^{Z_{r}}$, respectively. Here, *Z_r_* signifies the number of modes, typically taking values in the range of *Z_r_* = 5–10. To determine the lowest energy state of the conduction band and the highest energy state of the valence band in the MQW structure, the following expressions are utilized:

(16) $$E_{C}=-qV+\frac{1}{2}\left\{E_{g}+k_{B}T\;ln\left(\frac{N_{C}}{N_{V}}\right)\right\},$$

(17) $$E_{V}=E_{C}-E_{g}.$$

The space-dependent electric potential, denoted as *V*, is obtained as one of the solutions of the CLDD equations. *E_g_* represents the space-dependent bandgap, and *N_C_*_,*V*_ are the effective density states in the conduction and valence bands, respectively. The quantum hole and electron densities can then be computed using the following expressions:

(18) $$r^{\left(qunt\right)}=\sum_{Z_{r}}\left(N_{r}^{Z_{r}}{\left|\psi_{r}^{Z_{r}}\right|}^{2}\right),$$

where $N_{r}^{Z_{r}}$ denotes the sub-band hole and electron densities in the valence and conduction bands of the MQWs, respectively. These quantities are established through an understanding of the 2-D density-of-states function and Fermi–Dirac distribution functions associated with the hole and electron densities in the valence and conduction bands of the MQWs [31]. Ultimately, the space-dependent quantum-correction factors corresponding to the hole and electron densities are ascertained through the following expressions:

(19) $$\left.\begin{array}{c} \gamma_{r}=\left\{\frac{r^{\left(qunt\right)}}{r}\right\}\;\;\;\;\;\;\;\;\;\;\;\;\;\;\;in\;\Omega_{MQW}\\ \;\;\;\;\;\;\;\;\;=1\;\;\;\;\;\;\;\;\;\;\;\;\;\;\;\;\;\;\;\;\;\;\;\;\;\;\;Otherwise \end{array}\right\}.$$

By introducing the space-dependent parameter *γ_r_*, quantum corrections are incorporated into the hole (*p*) and electron (*n*) densities within the CLDD Equations (1)–(3) starting from the next iteration (iteration number ≥ 2). Following each iteration, the inter-iteration deviations of *V*, *n*, *p*, *J_n_*, and *J_p_* were individually computed [31]. Convergence to self-consistent solutions (*V*_(*SC*)_, $\xi_{\left(SC\right)}=-dV_{\left(SC\right)}/dz$, *n*_(*SC*)_, *p*_(*SC*)_, $J_{n\left(SC\right)}$ and $J_{p(SC)}$) was assumed to be reached when all inter-iteration deviations were found to be less than the order of 10^−3^ [31].

The avalanche zone width (*z_A_*_(*SC*)_) for the considered IMPATT structures was determined by finding the *z*-coordinate along the +*z*-axis at which the normalized current density reached $NJ\left(z=z_{A\left(SC\right)}\right)=+0.95$ for a given bias current density of $J_{0}=J_{p\left(SC\right)}+J_{n\left(SC\right)}$. Consequently, the drift zone width was given by *z_D_*_(*SC*)_ = (*H_n_* − *z_A_*_(*SC*)_). Essential DC parameters such as breakdown voltage (*V_B_*_(*SC*)_), avalanche zone voltage drop (*V_A_*_(*SC*)_), and drift zone voltage drop (*V_D_*_(*SC*)_) for a given current density (*J*_0_) can be derived from the following:

(20) $$V_{B\left(SC\right)}=-\int_{z=0}^{{z=H}_{n}}\xi_{\left(SC\right)}\left(z\right)dz,\;V_{A\left(SC\right)}=-\int_{z=0}^{{z=z}_{A\left(SC\right)}}\xi_{\left(SC\right)}\left(z\right)dz,\;\mathrm{and}\;V_{D\left(SC\right)}=V_{B\left(SC\right)}-V_{A\left(SC\right)}.$$

**Figure 4:**
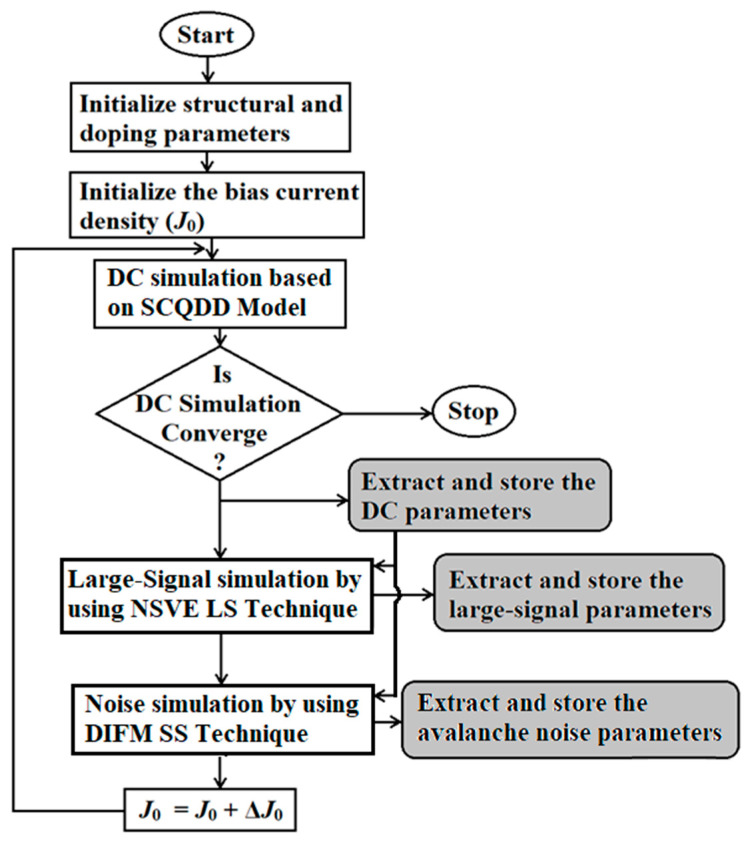
Flowchart illustrating the simulation technique.

Upon the successful convergence of the DC simulation for a given current density value (*J*_0_), various DC parameters, including avalanche zone width, drift zone width, avalanche voltage, drift voltage, and breakdown voltage, were extracted and stored. These parameters served as the initial conditions for subsequent large-signal simulation and small-signal noise simulation, which were conducted for the same *J*_0_. To perform the large-signal simulation for a given bias current density, the space and time-dependent Poisson’s equation, continuity equations, and current density equations were solved subject to the time-varying field and current boundary conditions [51]. A non-sinusoidal high-frequency voltage source (fundamental frequency is 1.0 THz) was introduced across the IMPATT device through appropriate capacitor-coupling in order to introduce THz oscillation in the large-signal formulation [51]. The large-signal solution provides time-domain waveforms of the diode terminal current (*i_d_*_(*sc*)_(*t*)) and voltage (*v_d_*_(*sc*)_(*t*)). The *i_d_*_(*sc*)_(*t*) and *v_d_*_(*sc*)_(*t*) waveforms are Fourier transformed using the appropriate fast Fourier transform (FFT) algorithm in order to obtain the corresponding frequency domains (*I_d_*_(*sc*)_(*f*) and *V_d_*_(*sc*)_(*f*)). The *I_d_*_(*sc*)_(*f*) and *V_d_*_(*sc*)_(*f*) are used to calculate the diode impedance (*Z_d_*_(*sc*)_(*f*) = *V_d_*_(*sc*)_(*f*)/*I_d_*_(*sc*)_(*f*) = *R_d_*_(*sc*)_(*f*) + *jX_d_*_(*sc*)_(*f*), where *R_d_*_(*sc*)_ and *X_d_*_(*sc*)_ are the diode resistance and reactance) and admittance, and (*Y_d_*_(*sc*)_(*f*) = 1/*Z_d_*_(*sc*)_(*f*) = *G*_(*sc*)_(*f*) + *jB*_(*sc*)_(*f*), where *G*_(*sc*)_ and *B*_(*sc*)_ are the diode conductance and susceptance) as functions of frequency for a given bias current density. The outcomes of the NSVE large-signal simulations encompassed a wide array of parameters such as diode impedance, admittance, negative conductance (*G*_(*sc*)_), susceptance (*B*_(*sc*)_), quality factor (*Q*_(*sc*)_ = –*B*_(*sc*)_/*G*_(*sc*)_), negative resistance (*R_d_*_(*sc*)_), reactance (*X_d_*_(*sc*)_), and series resistance (*R_s_*_(*sc*)_), all associated with the specified *J*_0_. These simulation results were carefully extracted and stored for further analysis and evaluation (Figure 4). Finally, the THz power output and the DC to THz conversion efficiency at a specific operating frequency (*f*) for a given current density (*J*_0_) were calculated using the following expressions:

(21) $$P_{THz\left(SC\right)}=\frac{1}{2}{\left(V_{THz\left(SC\right)}\right)}^{2}\left|G_{p\left(SC\right)}\right|A_{j},$$

(22) $$\eta_{L\left(SC\right)}=\frac{P_{THz\left(SC\right)}}{P_{DC\left(SC\right)}},$$

where *P_THz_*_(*sc*)_ represents the THz power output, which is proportional to the square of the root-mean-square value of the oscillating voltage at the fundamental THz frequency (*P_THz_*_(*sc*)_ ∞ (*V_THz_*_(*sc*)_/√2)^2^). This oscillating voltage (*V_THz_*_(*sc*)_) is given by the product of a modulation index (*m_x_*), typically ranging from 50% to 60%, and the breakdown voltage (*V_B_*_(*sc*)_). Additionally, |*G_p_*_(*sc*)_| denotes the magnitude of the peak negative conductance, and *A_j_* represents the junction area of the diode, calculated as *A_j_* = π*D_j_*^2^/4, with *D_j_* being the junction diameter. In Equation (22), *η_L_*_(*sc*)_ represents the DC to THz conversion efficiency, which is determined by the ratio of the THz power output (*P_THz_*_(*sc*)_) to the input DC power (*P_DC_*_(*sc*)_). The input DC power, denoted as *P_DC_*, can be expressed as the product of the current density (*J*_0_), breakdown voltage (*V_B_*_(*sc*)_), and the junction area (*A_j_*), i.e., *P_DC_*_(*sc*)_ = *J*_0_*V_B_*_(*sc*)_*A_j_*.

Following the completion of the large-signal simulation, a subsequent small-signal noise simulation was conducted using the double-iterative field-maximum small-signal (DIFM SS) noise simulation technique based on the SCQDD model [35]. The random impact ionization process introduces unpredictable fluctuations in the DC components of the particle current and electric field within the IMPATT device. These unanticipated fluctuations, occurring under reverse bias breakdown conditions, manifest as small-signal components relative to their corresponding steady-state DC values. For the small-signal avalanche noise simulation [52], an open circuit condition devoid of any high-frequency AC voltage signal was considered. The simulation involved the simultaneous solution of two second-order differential equations representing the real and imaginary parts of the noise field while adhering to appropriate boundary conditions. This computational approach employed the Runge–Kutta method and was designed to determine the noise field distribution along the depletion layer [53]. Notably, the band-to-band tunneling process was regarded as a noiseless instantaneous event, characterizing this noise simulation methodology as the DIFM method [52,53]. Ultimately, by leveraging knowledge about the mean square noise current and voltage, the transfer noise impedance at each spatial point along the depletion layer was computed. Subsequently, from the distribution of transfer noise impedance along the depletion layer, the mean square noise voltage $\langle v_{n}^{2}\rangle$ was derived. This information enabled the determination of the noise spectral density (*NSD*(*f*) = $\langle v_{n}^{2}\rangle /df$ V^2^ s) or mean square noise voltage per bandwidth (*df*) as a function of frequency (*f*). The assessment of the IMPATT source’s noise performance was accomplished through the utilization of a parameter known as the noise measure (*NM*(*f*)) [52,53] and defined as follows:

(23) $$NM\left(f\right)=\frac{NSD(f)}{4k_{B}T(-R_{d\left(SC\right)}(f){-R}_{s\left(sc\right)})}$$

Subsequent to the noise simulation for a given *J*_0_ value, critical noise parameters, such as noise spectral density (*NSD*(*f*)) and noise measure (*NM*(*f*)), were extracted and stored as functions of frequency for further analysis and evaluation.

**Figure 5:**
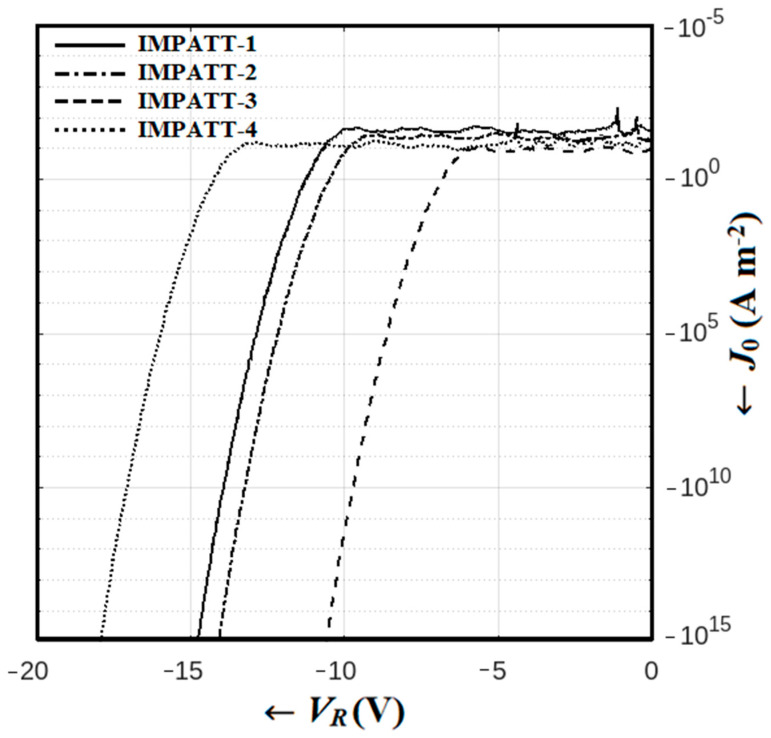
Reverse bias I–V characteristics of Al_0.3_Ga_0.7_N/GaN/Al_0.3_Ga_0.7_N MQW SDR (IMPATT-1), GaN SDR (IMPATT-2), Al_0.3_Ga_0.7_N/GaN/Al_0.3_Ga_0.7_N MQW Schottky barrier (IMPATT-3) and 3C-SiC/Si/3C-SiC MQW DDR (IMPATT-4) diodes.

Subsequently, the *J*_0_ value was systematically updated, and this iterative process was reiterated until the DC simulation no longer converged, thereby enabling the comprehensive assessment of the diode structure’s full-band THz-wave performance. A visual representation of the complete simulation methodology employed in this study is depicted in Figure 5. In addition, parasitic series resistance, stemming from the un-depleted epitaxial layers, anode and cathode ohmic contacts, and other un-depleted GaN contact-layers within the device, is meticulously calculated. This calculation is based on a previously reported method that takes into account the depletion width modulation phenomena [51] and also considers the influence of the skin effect [54].

The simulation incorporates the electric field dependency of material parameters such as the ionization rate (*α_n_*_,*p*_), drift velocity (*v_n_*_,*p*_), and mobility (*μ_n_*_,*p*_) of electrons and holes in GaN and Al_0.3_Ga_0.7_N, utilizing data from recent experimental studies [55,56,57,58,59]. Additionally, the influence of carrier mobility on doping concentration is factored in using established analytical models [59]. The valence and conduction band offsets (VBO and CBO) in GaN/Al_0.3_Ga_0.7_N heterojunctions are calculated from the empirical relations reported in ref. [60]. Other material parameters, including diffusion constant (*D_n_*_,*p*_), diffusion lengths (*L_n_*_,*p*_), high-frequency dielectric constants (*ε_s_*), relative permeabilities (*μ_s_*), effective masses (m^*^_n__,*p*_), etc., are sourced from recent experimental reports [61,62]. Parameters associated with *sp*- and *pz*-polarizations in GaN and Al_0.3_Ga_0.7_N are derived from published literature [49,50].

The work functions of nickel (Ni) at 5.15 eV and gold (Au) at 5.10 eV, along with the electron affinity of the Mg-doped *p*^+^-GaN layer, are crucial for forming high-quality anode ohmic contacts in IMPATT-1 and 2 structures. Conversely, the work functions of titanium (Ti) at 4.33 eV, aluminum (Al) at 4.28 eV, nickel (Ni) at 5.15 eV, and gold (Au) at 5.1 eV, along with the electron affinity of the Si-doped *n*^+^-GaN buffer layer, are essential for creating good-quality cathode ohmic contacts in IMPATT-1, 2, and 3 structures. Additionally, the work functions of nickel (Ni) at 5.15 eV and gold (Au) at 5.1 eV, along with the electron affinity of the *n*^+^-GaN layer (Si-doped), play a critical role in estimating the Schottky barrier height in the IMPATT-3 structure. While the electron affinity of undoped GaN is typically around 4.1 eV, the specific values of electron affinity (Χ*_e_*) are influenced by factors such as the doping level, temperature, and strain, particularly due to the relatively high doping concentrations of the Mg-doped *p*^+^-GaN and Si-doped *n*^+^-GaN layers compared to undoped GaN. These parameters are meticulously considered from published experimental data and integrated into the simulation of the diode structures to minimize computational errors [63,64].

## 4. Reverse Current–Voltage Characteristics

The DC simulation based on the SCQDD model employed a time-independent drift-diffusion model to acquire the DC characteristics of the diodes being studied. Figure 5 illustrates the reverse current–voltage (I–V) characteristics of the diodes, which were derived from the DC simulation. In the realm of semiconductor diode technology, the breakdown voltage, a critical parameter indicative of a diode’s ability to withstand high voltage without catastrophic failure, is a key consideration. In a comparative analysis of various diodes, the breakdown voltage emerges as a defining characteristic that significantly influences their performance. Firstly, we examined the characteristics of the GaN SDR (IMPATT-2) diode, which exhibited a breakdown voltage of 9.24 V. This value signifies the point at which the diode’s insulating properties breakdown, and it allows a current to flow freely in the reverse direction. On the other hand, the Al_0.3_Ga_0.7_N/GaN/Al_0.3_Ga_0.7_N MQW SDR (IMPATT-1) diode, incorporating wider bandgap barrier layers (Al_0.3_Ga_0.7_N layers) presents a slightly higher breakdown voltage of 10.07 V. This modest increase in breakdown voltage can be attributed to the presence of these barrier layers, which enhance the diode’s ability to withstand reverse voltage stress. Furthermore, IMPATT-1 showcases a smaller reverse leakage current, approximately 25 mA, in comparison to IMPATT-2, which will be discussed shortly. Speaking of IMPATT-1, its design incorporates wider bandgap barrier layers (Al_0.3_Ga_0.7_N layers), contributing to a slightly greater breakdown voltage than IMPATT-2. With a reverse leakage current on the order of 40 mA, IMPATT-1 showcases improved characteristics compared to IMPATT-2, thus demonstrating the influence of barrier layers on breakdown performance.

Now, we turn our attention to the 3C-SiC/Si/3C-SiC MQW DDR (IMPATT-4) diode, which stands out with the highest breakdown voltage of 13.42 V among the analyzed diodes. This impressive result can be attributed to the presence of two drift layers, *n*- and *p*-layers, in IMPATT-4, whereas the *p*-drift layer is absent in the single-drift diodes previously discussed. This additional *p*-drift layer contributes to the IMPATT-4 diode’s superior ability to withstand reverse voltage stress. However, it is noteworthy that the reverse leakage current of IMPATT-4 is comparatively higher, approximately 100 mA, which may be attributed to the combination of narrower bandgap barrier layers (3C-SiC) and a base material (Si) compared to IMPATT-1 and IMPATT-2. Lastly, the Al_0.3_Ga_0.7_N/GaN/Al_0.3_Ga_0.7_N MQW Schottky barrier (IMPATT-3) diode exhibits a significantly lower breakdown voltage of 6.44 V. Moreover, it displays the highest reverse leakage current, approximately 200 mA, among all the diodes studied. The highest leakage current in IMPATT-3 compared to the semiconductor *p^+^-n* or *p-n* junction diodes is primarily due to the presence of the TFE mechanism in the Schottky junction [47,48]. These limitations, notably the smaller breakdown voltage and larger reverse leakage current, are inherent to Schottky barrier IMPATT diodes in comparison to their counterparts based on *p-n* junction-based diodes (SDR and DDR IMPATT diodes).

In summary, the breakdown voltage and reverse leakage current are pivotal factors in assessing the performance and limitations of IMPATT diodes. The presence of wider bandgap barrier layers and multiple drift layers significantly influences the breakdown voltage, making it a key design consideration in the development of high-performance IMPATT diodes. Understanding these characteristics is crucial for selecting the appropriate IMPATT diode for specific applications and optimizing their performance THz regime.

## 5. Terahertz Characteristics 

**Figure 6:**
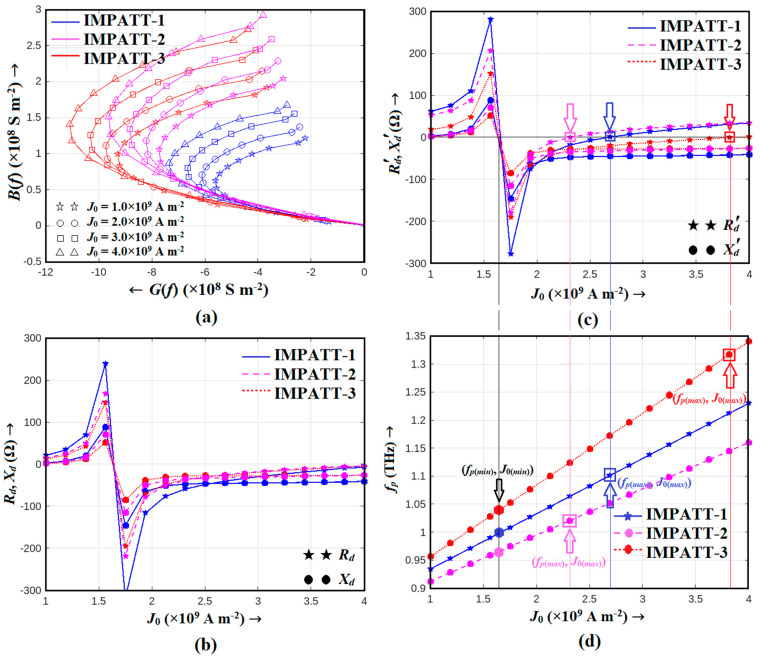
(**a**) Admittance characteristics of the diodes (IMPATT-1, IMPATT-2, and IMPATT-3) obtained from large-signal simulation without considering the effect of series resistance for different bias current densities ranging from 1.0 × 10^9^ to 4.0 × 10^9^ A m^−2^; (**b**) variations in diode resistance and reactance with bias current density obtained from the admittance characteristics without considering the effect of series resistance; (**c**) variations in diode resistance and reactance with bias current density obtained from the admittance characteristics by considering the effect of series resistance; (**d**) variations in optimum frequency corresponding to the peak magnitude of the negative conductance of the didoes with bias current density. The frequency ranges within which the diodes exhibit negative resistance (where *R_d_^’^* values are negative) are determined from (**c**) and the associated minimum (*f_p_*_(*min*)_) and maximum (*f_p_*_(*max*)_) frequencies of oscillation as well as corresponding bias current densities (*J*_0(*min*)_, *J*_0(*max*)_), which are highlighted in (**d**). From these minimum and maximum frequencies of oscillation, the oscillation bandwidth (*BW* = (*f_p_*_(*max*)_ − *f_p_*_(*min*)_)) of each source can be determined. The oscillation bandwidths of IMPATT-1, IMPATT-2, and IMPATT-3 sources were found to be 74.0, 46.5, and 264.0 GHz respectively.

In this study, a detailed analysis of diodes under consideration was conducted using the NSVE LS simulation technique based on the SCQDD model. Our investigation spanned a broad range of bias current densities, from 1.0 × 10^9^ to 4.0 × 10^9^ A m^−2^, to thoroughly explore the performance of IMPATT-1, IMPATT-2, and IMPATT-3 diodes. Initially, in Figure 6a, we present the admittance characteristics of these diodes without considering the impact of series resistance. Notably, the Al_0.3_Ga_0.7_N/GaN/Al_0.3_Ga_0.7_N MQW Schottky barrier diode, designated as IMPATT-3, exhibited a remarkable feat in terms of peak negative conductance (|*G_p_*|). Its |*G_p_*| values soared in the range of 0.9283 × 10^9^–1.1087 × 10^9^ S m^−2^, coinciding with an optimum frequency range of 1.02–1.18 THz. This exceptional performance highlighted IMPATT-3 as a standout candidate for THz applications. In stark contrast, IMPATT-1, the Al_0.3_Ga_0.7_N/GaN/Al_0.3_Ga_0.7_N MQW SDR diode, displayed the smallest |*G_p_*| values (0.5606 × 10^9^–0.7343 × 10^9^), while IMPATT-2, the GaN SDR diode, occupied an intermediate position (0.7727 × 10^9^–0.9624 × 10^9^) in this aspect.

Transitioning to Figure 6b, we delved into the diode resistance (*R_d_*) and reactance (*X_d_*) variations. These plots unveiled an avalanche resonance frequency, characterizing the point at which *R_d_* changed from positive to negative and *X_d_* shifted from an inductive to capacitive nature. This avalanche resonance phenomenon occurred within the narrow frequency range of 0.97–1.03 THz, which is a pivotal finding for our understanding of these diodes’ high-frequency capabilities. Remarkably, *R_d_* values remained negative for a bias current density range of 1.62 × 10^9^ to 4.0 × 10^9^ A m^−2^, underscoring the diodes’ suitability for THz frequency applications. To complete the picture, we ventured into estimating the series resistance of these diodes, which is a crucial parameter affecting their THz performance. Employing a method previously reported for IMPATT diodes and considering the influence of skin effect [20,21], we determined the series resistance ranges. IMPATT-1 exhibited series resistance within the range of 40.65–42.81 Ω, while IMPATT-2 fell within 37.87–39.18 Ω. Most notably, IMPATT-3, with its absence of metal–*p*^+^-GaN contact resistance due to the absence of the *p*^+^-GaN layer, demonstrated a series resistance as low as 4.34–5.67 Ω. This stark difference highlighted the inherent challenge posed by the high contact resistivity of the metal–*p*^+^-GaN ohmic contact (10^−4^–10^−3^ Ω cm^2^ [23,24,65]), especially for SDR diodes such as IMPATT-1 and IMPATT-2. In Figure 6c, we present the impedance characteristics of the diodes, taking into account the effect of series resistance. These curves provided valuable insights into the diodes’ behavior under realistic operating conditions. Simultaneously, Figure 6d showcases the relationship between the operating frequency and bias current density, which is a crucial parameter in understanding the dynamic performance of these diodes. The diodes’ negative resistance ranges were determined from Figure 6c, and this information enabled us to calculate the oscillation bandwidths (BW) of each source. IMPATT-1, IMPATT-2, and IMPATT-3 exhibited oscillation bandwidths of 74.0, 46.5, and an impressive 264.0 GHz, respectively. IMPATT-3′s significantly lower series resistance translated into an extended range of negative resistance, which, in turn, led to its commanding lead in terms of oscillation bandwidth.

**Figure 7:**
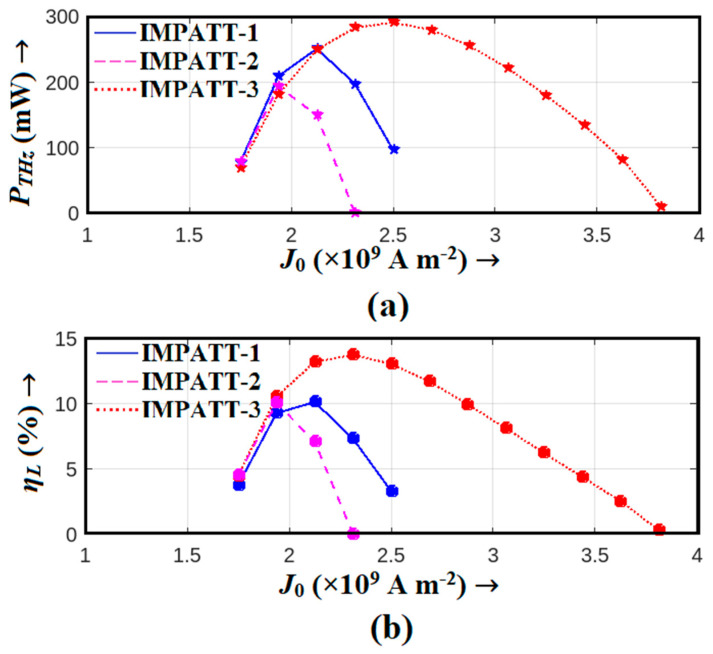
Variations in (**a**) THz power output and (**b**) large-signal efficiency of IMPATT-1, IMPATT-2, and IMPATT-3 sources with bias current density.

In our pursuit of THz performance, Figure 7a,b unveils the THz power output and DC to THz conversion efficiency versus bias current density. These figures offer a comprehensive view of how each source performed in real-world scenarios. Notably, the Schottky barrier (IMPATT-1) diode emerged as the frontrunner, achieving the highest THz power output of 290.87 mW and an impressive efficiency of 13.74% at an optimal bias current density of 2.5 × 10^9^ A m^−2^. IMPATT-2 and IMPATT-3 also displayed competitive performance, with peak THz power outputs of 251.0 and 193.8 mW and efficiencies of 10.13% and 10.07%, respectively, at their respective bias current densities (2.12 × 10^9^ and 1.94 × 10^9^ A m^−2^ respectively).

In summary, our comprehensive analysis of the diodes revealed that the Al_0.3_Ga_0.7_N/GaN/Al_0.3_Ga_0.7_N Schottky barrier diode, IMPATT-3, stood out as the most promising THz source. Its exceptional performance in terms of peak negative conductance, series resistance, and oscillation bandwidth underscores its potential for THz applications, positioning it as a leading candidate for THz power generation with impressive DC to THz conversion efficiency. These findings offer valuable insights for advancing THz IMPATT source technologies and their applications.

## 6. Avalanche Noise Characteristics

**Figure 8:**
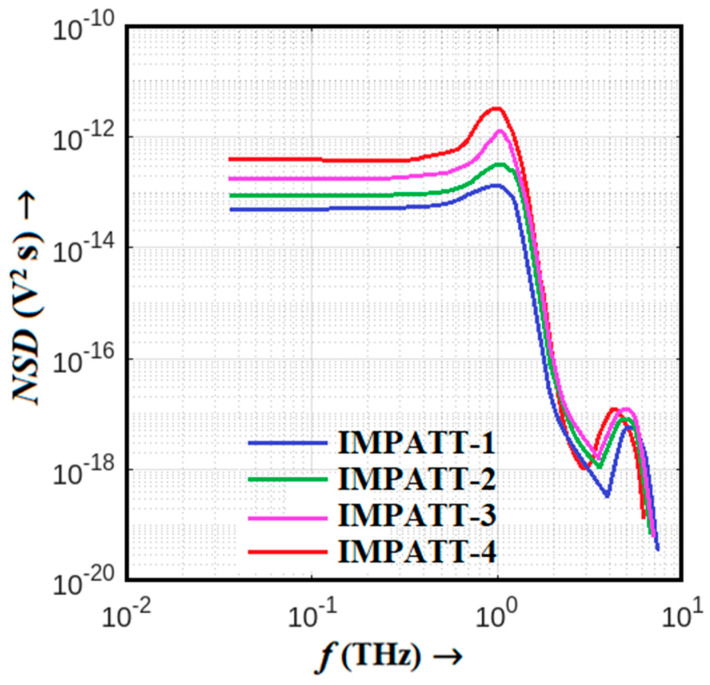
Variation in noise spectral density of the IMPATT-1, IMPATT-2, IMPATT-3, and IMPATT-4 THz sources with frequency; the inset of the figure illustrates an enlarged view of noise spectral density vs. frequency plots within the frequency range under consideration.

**Figure 9:**
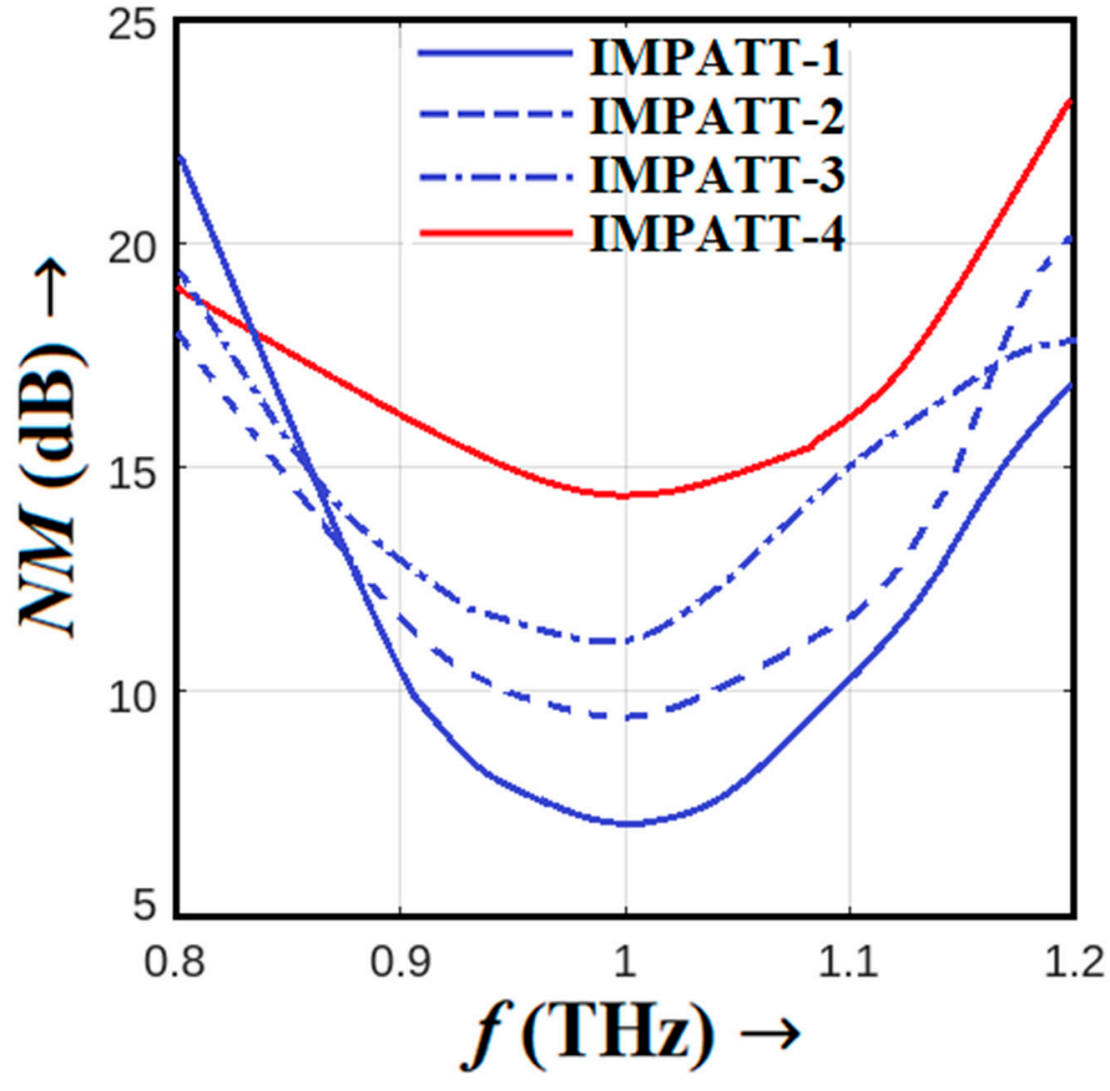
Variation in noise measurement of the IMPATT-1, IMPATT-2, IMPATT-3, and IMPATT-4 THz sources with frequency.

In the realm of Terahertz (THz) IMPATT sources, a thorough examination of their avalanche noise performance is crucial for understanding their operational characteristics. This analysis was conducted using the DIFM small-signal simulation technique based on the SCQDD model, as detailed in Section 3. The results of this study are presented graphically in Figure 8 and Figure 9, which depict the noise spectral density versus frequency graphs and noise measure versus frequency plots for the IMPATT sources under investigation. Figure 8 showcases the noise spectral density versus frequency for these sources, offering a visual representation of their noise performance across the THz spectrum. These data are instrumental in evaluating the noise characteristics of each source. The comparison between the different IMPATT sources reveals interesting insights. As a DDR diode, IMPATT-4 is characterized by a wider avalanche zone on both the *n*- and *p*-sides, combined with a narrower bandgap base material system, and exhibits the highest avalanche noise among its single-drift counterparts. 

This heightened noise is substantiated by a noise measure (NM) of 14.32 dB at 1.0 THz, as illustrated in Figure 9. In contrast, IMPATT-1, IMPATT-2, and IMPATT-3 display lower noise measurement values of 7.43, 9.52, and 11.75 dB, respectively. In the context of noise performance, smaller NM values are indicative of superior performance. The exceptional noise performance of IMPATT-1 can be attributed to the phenomenon of the quantum confinement of electrons in MQWs near the *p^+^-n* junction. This quantum confinement effectively narrows down the avalanche width, resulting in the best noise performance among the IMPATT sources studied. IMPATT-2, while still exhibiting good noise performance, shows a slightly wider avalanche region compared to IMPATT-1 due to the absence of MQWs. This broader avalanche zone introduces a larger amount of random impact ionizing collisions, impacting its noise performance negatively.

**Figure 10:**
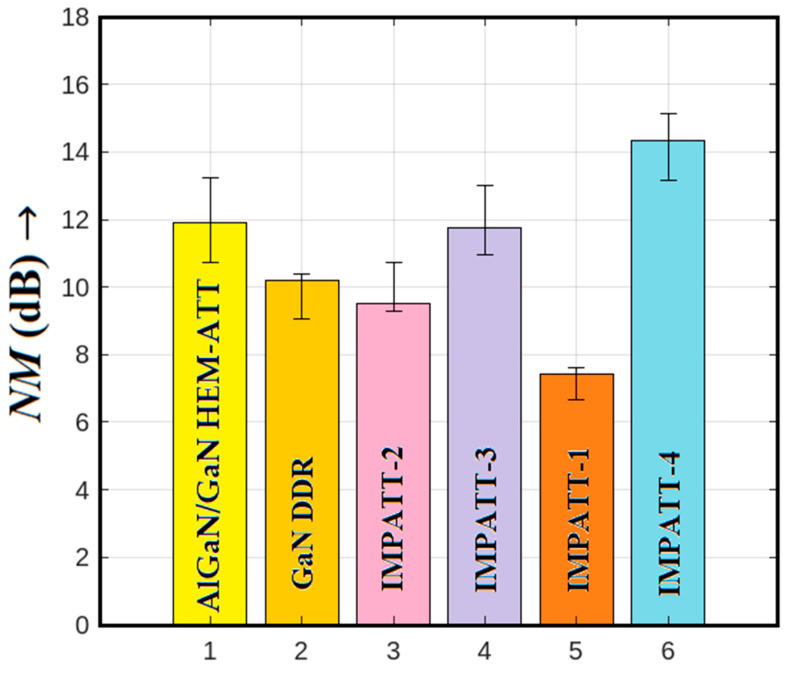
Bar charts illustrate the noise measure of AlGaN/GaN HEM-ATT [28], GaN DDR [28], IMPATT-1, IMPATT-2, IMPATT-3, and IMPATT-4 sources at 1.0 THz; the error bars represent the variation in the noise measure of a source within its frequency range of oscillation.

In the case of the IMPATT-3 source, which employs a Schottky barrier MQW SDR diode, carrier injection into the avalanche region occurs via the noisy thermo-ionic emission mechanism. While the quantum confinement of electrons in MQWs restricts the avalanche zone and limits avalanche noise to some extent, the inherent noise in IMPATT-3 is still increased. Consequently, its noise performance deteriorates compared to IMPATT-1 and IMPATT-2. The IMPATT-4 source, utilizing 3C-SiC/Si/3C-SiC MQW DDR technology, presents the worst noise performance among all the sources. The narrower bandgap barrier layers (3C-SiC) and base material (Si) result in the presence of larger amounts of thermally generated electron-hole pairs within the avalanche zone and thereby cause a higher likelihood of impact ionization collisions within the active region. This amplifies the random nature of impact ionization, leading to deteriorated noise performance. To visually summarize the noise performance at 1.0 THz, Figure 10 displays bar charts comparing the noise measurements of various sources, including AlGaN/GaN HEM-ATT [28], GaN DDR [28], IMPATT-1, IMPATT-2, IMPATT-3, and IMPATT-4. The error bars represent the variation in the noise measure within each source’s frequency range of oscillation. Notably, Al_0.3_Ga_0.7_N/GaN/Al_0.3_Ga_0.7_N MQW SDR (IMPATT-1) stands out as the source with the best noise performance among them all, highlighting the significance of quantum confinement effects in optimizing noise characteristics in THz IMPATT sources.

## 7. Comparison with Other THz Sources

**Figure 11:**
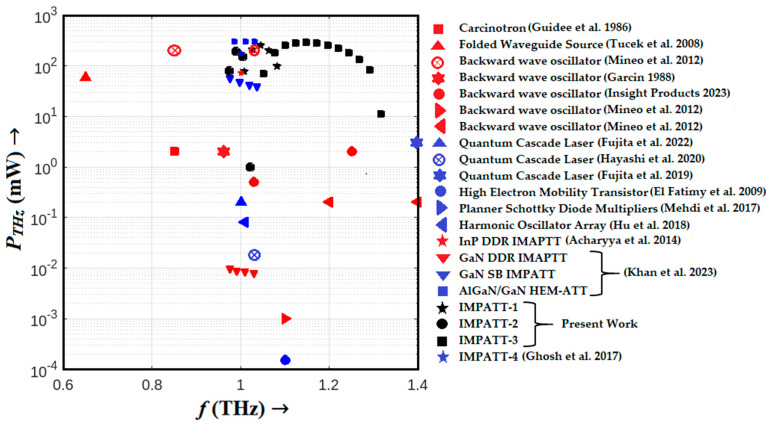
THz power output versus frequency plots of different THz sources (Refs. [1,2,3,4,5,6,7,8,9,10,11,21,28,31] and present work).

In this section, we present a comprehensive comparative analysis that juxtaposes various commercially available state-of-the-art THz oscillators, all operating at approximately 1.0 THz, with our proposed THz IMPATT sources based on the AlGaN/GaN/AlGaN MQW architecture. To facilitate a clear comparison, we graphically depicted the THz power output of these sources as a function of frequency in Figure 11 [1,2,3,4,5,6,7,8,9,10,11,28]. The key specifications, including the frequency range of operation, peak THz power output, and the DC to THz conversion efficiency of established state-of-the-art THz sources, such as the carcinotron, folded waveguide source, backward wave oscillators (BWOs), quantum cascade lasers (QCLs), high electron mobility transistors (HEMTs), planar Schottky barrier diode multipliers, and harmonic oscillator arrays, are listed in a tabular format in ref. [28]. Within the frequency range of 0.85–1.03 THz, our comparative analysis identifies the BWO with a narrow-corrugated waveguide slow wave structure, as fabricated and tested by Mineo et al. in 2012 [3], as the most promising source for THz generation. Their pioneering work achieved a noteworthy RF power output of 200 mW, demonstrating significant prowess within the specified tuning range. It is important to note that the remaining devices reviewed in our analysis exhibited comparatively lower THz power outputs, typically ranging from a few µW to mW, and relatively modest conversion efficiencies, often falling below 1.0% at THz frequencies. In stark contrast, the Schottky barrier low–high–low HEM-ATT source proposed by Khan et al. [28] emerges as a formidable contender, capable of delivering significantly higher THz power (~300 mW) along with commendably higher DC to THz conversion efficiency (11%) within the frequency range of 0.923–1.066 THz, encompassing an impressive oscillation bandwidth of 143 THz. Notably, this represents a substantial advancement compared to the performance of established THz sources. 

However, the pinnacle of our findings is the Al_0.3_Ga_0.7_N/GaN/Al_0.3_Ga_0.7_N MQW Schottky barrier IMPATT source, which surpasses all other potential THz sources, including the HEM-ATT source, in terms of THz power delivery (~300 mW), DC to THz conversion efficiency (~13%) and the bandwidth of oscillation (264 GHz). This exceptional performance can be attributed to the unique characteristics of the Al_0.3_Ga_0.7_N/GaN/Al_0.3_Ga_0.7_N MQW structure, which not only enhances THz power generation but also bolsters avalanche noise performance at THz frequencies. These attributes collectively position the Al_0.3_Ga_0.7_N/GaN/Al_0.3_Ga_0.7_N MQW Schottky barrier IMPATT source as the frontrunner for future THz applications. 

Furthermore, unlike double-drift region (DDR) GaN IMPATT diodes, the proposed Al_0.3_Ga_0.7_N/GaN/Al_0.3_Ga_0.7_N MQW Schottky barrier IMPATT structure exhibits significantly low parasitic series resistance due to the absence of a *p*-layer. In DDR GaN IMPATT diodes, the series resistance increases notably due to the large metal–*p*^+^-GaN anode ohmic contact, as the contact resistivity of state-of-the-art metal–*p*^+^-GaN ohmic contacts is considerably high compared to existing metal–*n*^+^-GaN ohmic contacts [23,24]. This high series resistance leads to very low negative resistance in DDR GaN diodes at sub-millimeter-wave and THz regimes. In the proposed MQW structure, the metal–*p*^+^-GaN anode contact was completely eliminated by employing the Ni/Au–*n*^+^-GaN Schottky anode contact, resulting in very low series resistance and significantly improved THz performance.

The proposed avalanche transit time diodes offer distinct advantages over other THz sources, such as the carcinotron, folded waveguide source, and BWOs, for THz radiation generation. Notably, our Al_0.3_Ga_0.7_N/GaN/Al_0.3_Ga_0.7_N MQW Schottky barrier IMPATT diode operated efficiently at remarkably low voltage levels, typically in the range of 5–7 V, to achieve breakdown under operational conditions. In contrast, existing THz sources often require significantly higher cathode voltages, often in the order of kilovolts (KV), to become operational. Additionally, traditional THz sources like the carcinotron, folded waveguide source, and BWOs tend to be bulky [1,2,3,4,5], while the MQW IMPATT oscillators, with their waveguide cavity and biasing arrangements, are notably less bulky and more compact. Furthermore, tuning the operating frequency and power in IMPATT oscillators via externally applied optical energy or magnetic fields is much more convenient compared to other sources relying on electronic-oscillation principles [66,67,68,69]. In contrast to photonic THz sources like QCLs, which require very low cryogenic temperatures to function effectively, maintaining such conditions demands additional arrangements and consumes significant electric power during operation [6,7,8]. Conversely, IMPATT diodes can operate at room temperature without the need for cryogenic cooling. This simplifies system design and operation, reducing overall complexity and costs. Moreover, the simpler structure of IMPATT diodes facilitates a much simpler fabrication process compared to complex devices like HEMTs, planar Schottky diode multipliers, and harmonic oscillator array structures. This simplicity streamlines its fabrication and integration into various systems, making IMPATT sources appealing for commercial applications where cost efficiency is paramount. This notable difference highlights the superior potential and reliability of our Schottky barrier MQW IMPATT sources in the field of THz technology.

In summary, our comparative study illuminates the remarkable performance of the Al_0.3_Ga_0.7_N/GaN/Al_0.3_Ga_0.7_N Schottky barrier IMPATT source and highlights its significant advantages over existing THz sources [1,2,3,4,5,6,7,8,9,10,11]. Its combination of superior THz power output, enhanced conversion efficiency, and low operational voltage requirements firmly establishes it as an innovative and reliable THz source, poised to advance THz technology and its applications. Due to the challenges in fabricating and characterizing THz IMPATT oscillators based on the AlGaN/GaN material system discussed in this paper, experimental reports on these THz sources are currently unavailable in the published literature. While some researchers have recently attempted to experimentally realize SDR IMPATT sources based on GaN at lower microwave frequencies [70,71,72], the more thorough investigations presented in this paper offer significant potential for the realization of THz AlGaN/GaN/AlGaN MQW IMPATT sources in the near future.

## 8. Conclusions

In this research endeavor, we explored the potential of edge-terminated SDR MQW IMPATT structures based on the GaN/Al_0.3_Ga_0.7_N material system to be applied to high-power THz wave generation. Our investigation centered on the following two primary MQW diode configurations: (i) *p^+^-n* junction-based and (ii) Schottky barrier diode structures, both aimed at harnessing their THz capabilities. Furthermore, we proposed mesa etching in conjunction with nitrogen ion-implantation techniques for effective edge termination, successfully ameliorating premature and soft breakdown issues that previously plagued GaN diodes. Through an extensive examination encompassing steady-state and high-frequency THz characterizations, we employed an indigenously developed steady-state and NSVE large-signal simulation platform grounded in the earlier developed, well-established SCQDD model. Our simulations also extended to evaluate the steady-state and high-frequency THz performances of SDR IMPATT and DDR IMPATT diodes, utilizing both GaN and 3C-SiC/Si/3C-SiC MQW material systems for comparison. The outcomes of our research reveal the pivotal role of Schottky barriers in significantly diminishing device series resistance, consequently leading to substantial enhancements in peak continuous wave (CW) power output, achieving approximately 300 mW, and DC to THz conversion efficiency, nearing 13% at an operational frequency of 1.0 THz. This outcome underscores the tremendous promise of Schottky barriers in the realm of THz technology. In addition, we ventured into the arena of noise performance, utilizing a DIFM small-signal noise simulation technique rooted in the SCQDD model. Our investigation into the avalanche noise characteristics of the proposed THz sources revealed that the inclusion of MQW structures within the diode’s avalanche zone constitutes a significant noise mitigation strategy, significantly enhancing overall device performance under realistic operating conditions. To affirm the superiority of our proposed THz sources, we conducted a comparative analysis against several state-of-the-art THz sources documented in the literature. This rigorous benchmarking exercise decisively established the exceptional capabilities of our proposed THz sources. In summary, our study not only advances the understanding of THz technology but also underscores the potential for Al_0.3_Ga_0.7_N/GaN/Al_0.3_Ga_0.7_N MQW Schottky barrier IMPATT structures to become leading candidates for high-power THz wave generation. These findings hold great promise for the evolution of THz technology and its myriad applications across diverse scientific and industrial domains.

## Data Availability

Data are contained within the article.
